# Beyond pain relief: the effects of chronic opioid use on brain structure and function in diabetic neuropathy—a multimodal neuroimaging study

**DOI:** 10.1007/s00125-025-06529-w

**Published:** 2025-10-06

**Authors:** Gordon Sloan, Kevin Teh, Marni Greig, Pallai Shillo, Sharon Caunt, Iain D. Wilkinson, Solomon Tesfaye, Dinesh Selvarajah

**Affiliations:** 1 Diabetes Research Unit, Royal Hallamshire Hospital, Sheffield Teaching Hospitals NHS Foundation Trust, Glossop Road, Sheffield, UK; 2 Division of Clinical Medicine, University of Sheffield, Sheffield, UK

**Keywords:** Diabetic neuropathy, Opioids, Painful diabetic neuropathy, Resting-state functional MRI

## Abstract

**Aims/hypothesis:**

Despite being commonly prescribed to treat painful diabetic peripheral neuropathy (DPN), the impact on the brain of long-term opioid use as analgesia is unknown. The aim of this study was to determine the structural and functional brain alterations associated with prescription opioid use in a large cohort of people with painful DPN.

**Methods:**

A total of 82 patients with diabetes were enrolled: 57 with painful DPN (18 with long-term opioid prescription [O+ individuals] and 39 who were not prescribed opioids [O− individuals]) and a control group of 25 patients with diabetes but without DPN (no DPN) matched for age (± 2 years), sex and type of diabetes. All participants underwent detailed clinical/neurophysiological assessment and brain MRI at 3 T, and a subset (14 in each group, *n*=42) also underwent resting-state functional MRI.

**Results:**

O+ individuals had greater caudate volume (ANOVA, *p*=0.03) compared with O− individuals (*p*=0.03) and those with no DPN (*p*=0.01). Functional connectivity was lower between the caudate and thalamus (*r* β = −0.24, seed-level correction −3.9, *p*_FDR_ ≤0.05) in O+ individuals compared to those with no DPN. Moreover, seed-to-voxel analysis using caudate as the seed showed a significantly lower functional connectivity in O+ individuals compared with O− individuals in a cluster encompassing the superior frontal gyri bilaterally.

**Conclusions/interpretation:**

We demonstrate that disruption of dopaminergic pathways occurs within the brain when opioids are used for analgesic purposes for painful DPN, which may reflect alterations in reward systems. This study has important clinical implications, as the measures of dopaminergic pathways found in this study may represent neuroimaging biomarkers that could be used to diagnose and monitor the negative consequences of prescription opioid use.

**Graphical Abstract:**

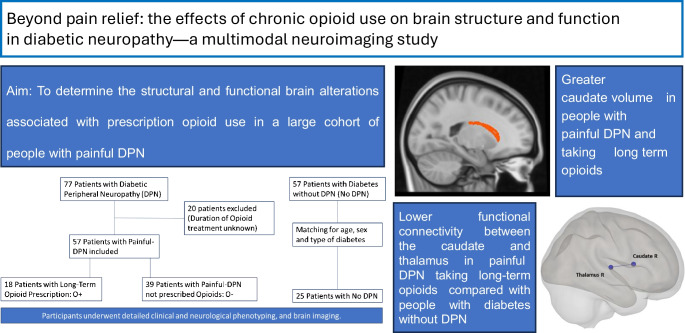

**Supplementary Information:**

The online version contains peer-reviewed but unedited supplementary material available at 10.1007/s00125-025-06529-w.


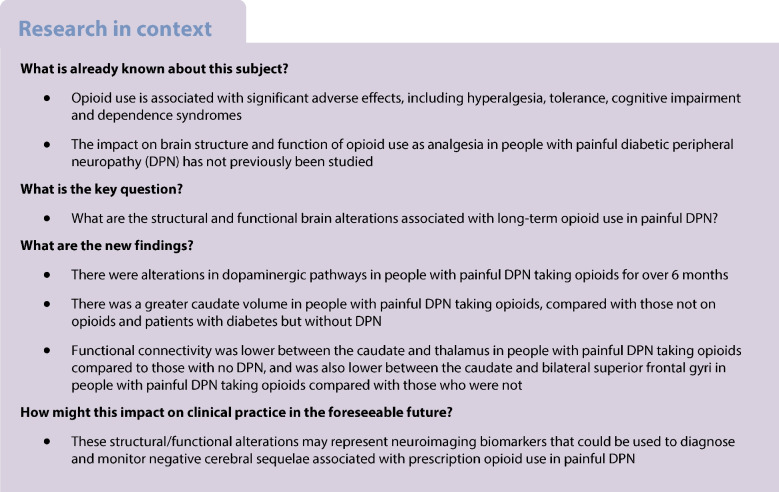


## Introduction

Painful diabetic peripheral neuropathy (painful DPN) is one of the most common chronic pain conditions across the world, occurring in up to a third of people with diabetes [1]. The treatment of painful DPN, like other chronic pain conditions, is inadequate, with only approximately half of people attaining 50% pain relief with common first-line treatments [2]. As a result, people with painful DPN are often prescribed opioid analgesics. Consistent with recommendations for other chronic pain conditions [3–6], guidelines for treatment of painful DPN either recommend against the use of opioids or recommend their use as third-line agents [7]. Despite this, opioid prescriptions worldwide continue to rise [8], with recent data showing that 40% of all patients with painful DPN are prescribed opioids [9]. Despite the well-known sequelae of prescription opioids, with soaring rates of opioid prescription-related drug abuse, overdose, addiction and death [6], the consequences of opioid prescription in painful DPN have not been investigated.

The biological adverse effects associated with opioid use include hyperalgesia, tolerance and drowsiness, and impaired memory, concentration and judgement [4]. Moreover, opioids often lead to dependence syndromes such as opioid use disorder or addiction, and withdrawal symptoms on discontinuation of treatment. Despite their continued widespread prescription, there is a lack of understanding of how these medications alter brain structure and function, particularly in the context of long-term prescription for pain. Such knowledge could be crucial, as it may be possible to use cerebral markers associated with opioid prescription to optimise existing therapeutic approaches [10]. However, only a few small studies have explored brain alterations associated with opioid prescription in chronic pain [11–13].

Therefore, there is a clear rationale to examine the structural and functional brain alterations associated with opioid prescription in people with painful DPN. In this study, we have used a cohort that previously demonstrated significant structural alterations in key somatomotor/nociceptive brain regions in patients with painful DPN [14]. This large neuroimaging database of well-phenotyped patients provides a unique opportunity to investigate the impact of long-term opioid use on the brain, and includes matched disease control patients with diabetes but without DPN. Moreover, a subset of these participants have also undergone resting-state functional MRI (rs-fMRI), allowing us to determine the functional connectivity between various regions of the brain. Our aim was to determine the structural and functional brain alterations associated with long-term opioid use for painful DPN.

## Methods

The participants included in the present database analysis were previously enrolled in a cross-sectional, observational, case–control cohort study of 283 right-handed individuals (217 with diabetes and 66 healthy volunteers) recruited from outpatient diabetes clinics at the Royal Hallamshire Hospital (Sheffield, UK) between 2009 and 2019, who are representative of the patients with diabetes under secondary care in the region [14]. Ethnicity data were collected and are reported, and the sex of participants was determined by their legal sex recorded at birth based on their biological characteristics. Inclusion criteria for the study were: right-handedness, age 18–85 years, and type 1 or type 2 diabetes diagnosed >6 months previously, and fulfilling the criteria for painful DPN or a diagnosis of diabetes without DPN (no DPN). Exclusion criteria were pregnancy, insufficient command of the English language or insufficient mental capacity to provide informed consent, concurrent severe psychological/psychiatric conditions, moderate to severe pain from causes other than DPN, non-diabetic neuropathies (e.g. thyroid disease, vitamin B_12_ or folate deficiencies, drug-induced or toxic neuropathy, or inflammatory, autoimmune or genetic neuropathy), other diabetic neuropathies (e.g. lumbosacral plexopathy, mononeuropathies), history of alcohol consumption >20 units/week (1 unit equivalent to one glass of wine or one measure of spirits), current or historical recreational drug abuse or addiction (including alcohol), recurrent severe hypoglycaemia, neurological disorders that may confound radiological or clinical assessments (e.g. cerebrovascular disease, epilepsy, dementia, multiple sclerosis), contraindications to MRI (e.g. pacemaker, claustrophobia) and opioid use in patients without painful DPN. All participants gave written informed consent before participating in the study, which had prior ethics approval from the NHS Health Research Authority (Sheffield, UK) review board.

### Participant assessment

Study group participants underwent detailed clinical history, neurological examination and biochemical assessments. Neurophysiological testing included nerve conduction studies, performed at a stable skin temperature of 31°C and a room temperature of 24°C using a Medelec electrophysiological system (Synergy Oxford Instruments, Oxford, UK). The following nerve attributes were measured: (1) sural sensory nerve action potentials and conduction velocities; (2) common peroneal distal latency, compound muscle action potential and conduction velocity; and (3) tibial motor nerve distal latency.

The presence of painful DPN was confirmed on the basis of meeting all of the following criteria: (1) the American Academy of Neurology minimum case definition criterion to confirm the presence of DPN (i.e. an abnormality [>99th or <1st percentile] of any attribute of nerve conduction in two separate nerves, one of which must be the sural nerve [15]); (2) a Douleur Neuropathique 4 (DN4) score >4 [16]; and (3) neuropathic pain diagnosed according to the International Association for the Study of Pain definition for a duration of 6 months or greater [17].

Patients with painful DPN were divided into two subgroups based on neurophysiological assessments and medication history: (1) painful DPN with long-term opioid prescription, defined as prescription for >6 months (O+ individuals); and (2) painful DPN with no evidence of opioid prescription within the last 12 months (O− individuals). Patients with DPN without neuropathic pain (i.e. painless DPN) and those with a prescription of opioids for <6 months or an undefined duration were not included in either group. A control group (*n*=25) of diabetic patients with no DPN (diabetes in the absence of DPN or neuropathic pain) matched for age (± 2 years), sex and type of diabetes was included in the analysis.

Quantitative sensory testing (QST) was performed according to the German Pain Research Network on Neuropathic Pain protocol [18]. The following QST parameters were measured: (1) cold and warm detection threshold (CDT and WDT) and thermal sensory limen (TSL) using a TSA-II neurosensory analyser (Medoc, Ramat Yishai, Israel); (2) thermal pain thresholds for cold and hot stimuli (CPT and HPT); (3) mechanical pain sensitivity (using a cotton wool ball, cotton bud and paintbrush, and 8, 16, 32, 64, 128, 256 and 512 mN metal probes [MRC Systems, Heidelberg, Germany]), including thresholds for pinprick (mechanical pain threshold [MPT]) and blunt pressure (pressure pain threshold [PPT]) (using an Algometer [Somedic, Sösdala, Sweden]), and stimulus/response functions for pinprick sensitivity (mechanical pain sensitivity [MPS]) and dynamic mechanical allodynia (DMA); and (4) the mechanical detection threshold (MDT) (using standardised von Frey filaments of 0.25, 0.5, 1, 2, 4, 8, 16, 32, 64, 128 and 256 mN [Nervtest, Marstock, Germany]) and vibration detection threshold (VDT) using a tuning fork (64 Hz, 8/8 scale).

The severity of neuropathic pain was assessed using an 11-point visual analogue scale (where 0 = no pain and 10 = worst pain imaginable).

### MRI acquisition and analysis

The magnetic resonance brain scan was performed at 3 T, with all participants having a three-dimensional T1-weighted magnetisation-prepared rapid echo sequence for anatomical data, and a subset of participants undergoing a 6 min rs-fMRI sequence acquired while participants fixated on a cross using a T2*-weighted pulse sequence. Further details of scan acquisition and MRI analysis are provided in the electronic supplementary materials [ESM] Methods. Imaging was performed when participants had discontinued analgesics for at least 48 h to minimise the potential confounding effects of pain relief on imaging measures.

Measurement of cortical thickness and global and deep brain nuclei quantification were performed using FreeSurfer software (https://surfer.nmr.mgh.harvard.edu) [14]. Global brain volume quantification was performed for total brain volume, cortical volume, total cortical white matter volume, subcortical grey volume and total grey volume. Regions of interest (ROI) were chosen in regions related to somatomotor function (primary somatosensory and motor cortex, insular cortex, anterior cingulate gyrus, thalamus) and key regions involved in dependence (putamen, amygdala, nucleus accumbens and caudate nucleus). ROI volumetric data from each hemisphere were combined prior to statistical analysis.

Subcortical structures identified as having a group difference in FreeSurfer analysis were further analysed using FSL-FIRST. FSL-FIRST is an automated model-based segmentation/registration tool within the FMRIB software library, version 6.0.3. FSL-FIRST was used according to prior guidelines [19]. Images were initially registered to the MNI152 standard space template, and registration was visually checked for each participant. FSL-FIRST uses a training data‐based approach and a Bayesian probabilistic model to determine the most probable shape of subcortical structures given the intensities of the T1 image. Surface meshes of the subcortical ROI were converted to boundary-corrected volumetric representations and boundary correction was automatically generated using the ‘run_first_all’ command line. Successful segmentation of ROI was visually verified, and masks were extracted into separate files from the single image containing the ROI labels. Our group comparison investigated differences between O+ individuals and O− individuals. Vertex shape analysis was applied using 5000 permutations, and significance was defined as *p*<0.05.

Next, we performed a more detailed analysis of cortical brain structure using a widely applied model for assessing brain morphology by quantifying and comparing the relative concentrations of grey matter throughout the brain between the two groups. This analysis is performed using a voxel-by-voxel-based method, voxel-based morphometry, within the FMRIB software library, version 6.0.3. Our group comparison investigated differences between O+ individuals and O− individuals, O+ individuals and those with no DPN, and O− individuals and those with no DPN. Voxelwise general linear modelling was then applied using permutation-based non-parametric testing (5000 permutations). Clusters of significance were identified using the threshold-free cluster enhancement method [20], with a family-wise error rate-corrected *p* value <0.05. The family-wise error multiple comparison correction is based on the Bonferroni method and controls the likelihood of false-positive findings in analyses.

rs-fMRI analysis was performed using the NITRC functional connectivity toolbox (CONN version 18.b) [14] and SPM12 (Wellcome Trust Centre for Neuroimaging, London, UK) in MATLAB version 2021a (The MathWorks, Natick, MA, USA) in a matched number of individuals in each group. The initial rs-fMRI analyses were carried out using ROI-to-ROI analysis. This approach measures correlations between ROIs within the default mode network, the thalamus, caudate, putamen, amygdala, nucleus accumbens, cingulate gyri, insular cortices, precentral and postcentral gyri, and frontal orbital cortices. Further seed-to-voxel analysis was performed on the seed selected based on structural and ROI-to-ROI rs-fMRI abnormalities. False discovery rate (FDR) correction was applied using the Benjamini–Hochberg method, implemented in the CONN toolbox. For ROI-to-ROI analysis, FDR correction was applied at the seed level; for seed-to-voxel analysis, it was applied across all brain voxels.

### Statistical analysis

Values for continuous baseline characteristics are presented as means ± SD and those for categorical variables are presented as the number and percentage. Differences in group variables were compared using ANOVA (continuous data, presented with 95% CI) or the χ^2^ test (categorical variables). Subgroup comparison for the severity of neuropathic pain and regional cortical and subcortical morphometric measurements between O+ individuals and O− individuals was performed using a two-tailed unpaired independent *t* test. The relationships between structural neuroimaging and clinical/neurological variables were assessed in more detail using Pearson correlation coefficients. All statistical analyses were completed using SPSS software, version 28.0 (SPSS Statistics for Windows [IBM, USA]).

## Results

### Participant group assignment

**Fig. 1:**
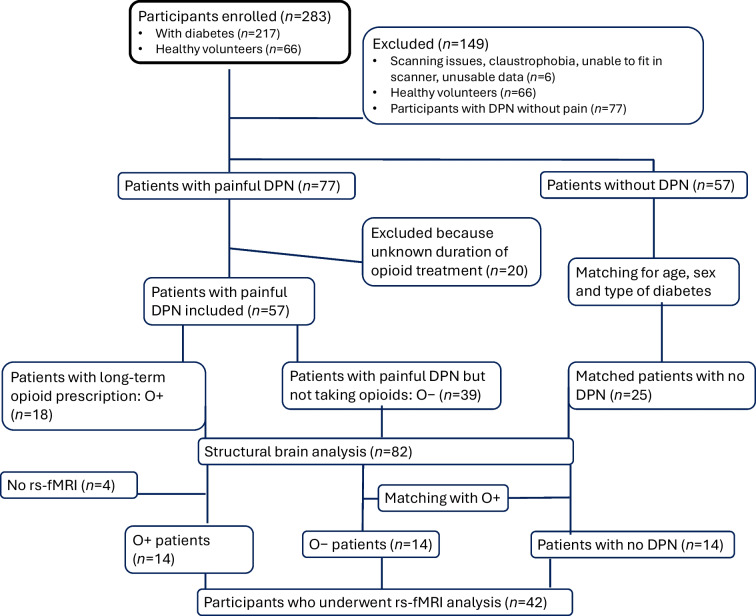
Flow diagram

A total of 77 participants with painful DPN and 57 participants with no DPN were recruited. Of the of 77 participants with painful DPN, 18 were designated as O+ individuals and 39 were designated as O− individuals; 20 patients with painful DPN were excluded due to missing data regarding analgesics prescribed, opioid prescription for <6 months, or opioids prescribed within the last year but discontinued. After matching for age, sex and type of diabetes, 25 patients with no DPN were included, leaving a total sample size of 82 (Fig. 1). Participants were predominantly White British, with two African British participants, both of whom were in the no DPN group.

### Participant assessments

Demographic characteristics and the results of clinical and neurological assessments are shown in Table 1. There were no group differences for age, sex and type of diabetes. The duration of diabetes was longer in O+ individuals (ANOVA, *p*<0.01) compared with O− individuals (*p*=0.01, 95% CI 2.0, 13.5) and those with no DPN (*p*<0.01, 95% CI 3.7, 16.1). The BMI was greater in O+ individuals (ANOVA, *p*=0.05) compared to those with no DPN (*p*=0.04, 95% CI 0.2. 7.8), and in O– individuals compared to those with no DPN (*p*=0.03, 95% CI 0.5, 6.8). HbA_1c_ was greater in O+ individuals (ANOVA, *p*=0.03) compared with O− individuals (*p*=0.03, 95% CI 0.9, 23.6) and those with no DPN (*p*=0.01, 95% CI 4.2, 28.7).

**Table 1:** Demographic characteristics and the results of metabolic and neurophysiological assessments for each study cohort

Variable	O+ individuals (*n*=18)	O− individuals (*n*=39)	No DPN (*n*=25)	*p* value
Age, years	58.5±10.3	60.4±8.5	59.3±7.1	0.57
Female	5 (27.8)	12 (30.8)	8 (32.0)	0.96^a^
Type 1 diabetes mellitus	5 (27.8)	4 (10.3)	3 (12.0)	0.20^a^
Duration of diabetes mellitus, years	20.8±12.7	13.0±9.0	10.8±9.8	<0.01
BMI, kg/m^2^	32.6±5.6	32.2±7.4	28.6±4.2	0.05
HbA_1c_, mmol/mol	77.9±19.3	65.6±19.9	61.5 ±20.5	0.03
HbA_1c_, %	9.3±1.8	8.2±1.8	7.8±1.9	0.03
Smoking status^c^, %				
Current	25.0	24.2	4.5	0.64^a^
Ever smoked	68.8	54.5	59.1	0.13^a^
Alcohol intake				
Any alcohol use, %	33.3	33.3	50.0	0.38
Units/week	3.31±6.2	2.9±5.4	5.0±5.8	0.38
Peroneal velocity, m/s	34.7±6.2	37.4±5.5	44.6±4.5	<0.01
Peroneal latency, ms	6.9±3.0	6.7±3.3	4.8±0.9	0.02
Peroneal amplitude, mV	1.5±2.0	1.7±2.2	6.0±2.3	<0.01
Tibial latency, ms	9.9±8.9	7.1±2.7	4.4±0.5	0.01
Severity of neuropathic pain^d^	8.1±1.4	5.5±2.8		<0.01^b^
QST parameters, *z* score				
CDT	−2.8±0.7	−2.3±0.9	−2.0±3.4	0.44
WDT	−1.9±0.5	−1.8±0.4	−0.5±0.9	<0.01
TSL	−2.5±0.8	−2.1±0.6	−0.9±1.0	<0.01
CPT	−0.9±0.6	−0.9±0.4	−0.4±0.9	<0.01
HPT	−1.4±0.6	−1.4±0.4	0.2±1.6	<0.01
PPT	0.5±4.8	−0.2±2.2	0.7±1.3	0.39
MPT	−1.8±1.3	−1.4±1.8	0.7±1.4	<0.01
MPS	−0.9±1.5	−0.7 ±1.8	0.0 ±1.3	0.11
MDT	−3.7±1.4	−2.4±1.5	0.6±1.3	<0.01
VDT	−3.1±2.0	−3.1±2.3	−0.4 ±1.3	<0.01
DMA	4 (22.2)	5 (12.8)	1 (4.0)	0.20^a^
Neuropathic pain treatments^e^				
TCAs	4 (22.2)	6 (15.4)		0.71^a^
SNRIs	6 (33.3)	10 (25.6)		0.55^a^
Anticonvulsants	7 (38.9)	24 (61.5)		0.16^a^
Type of opioid				
Codeine	3 (16.7)			
Tramadol	5 (27.8)			
Morphine MR	1 (5.6)			
Buprenorphine	1 (5.6)			
Combination^f^	5 (27.8)			
Unspecified	3 (16.7)			

Data are presented as means ± SD for continuous data and percentage or *n* (%) for categorical data

Groups were compared using ANOVA, unless otherwise indicated: ^a^χ^2^ test; ^b^*t* test

^c^Missing data: *n*=11

^d^Measured using an 11-point visual analogue scale (where 0 = no pain and 10 = worst pain imaginable)

^e^Some patients receiving more than one neuropathic pain agent

^f^Any opioids used in combination with one another

MR, modified release; SNRIs, serotonin noradrenaline (norepinephrine) re-uptake inhibitors; TCAs, tricyclic antidepressants

As expected, all nerve conduction measures were significantly lower in the O+ individuals and O− individuals compared to those with no DPN. There were no statistically significant differences between the two painful DPN groups. For the QST parameters, WDT, TSL, CPT, HPT, MPT and VDT were significantly lower in O+ individuals and O− individuals compared with no DPN, but there were no significant differences between the painful DPN groups. The MDT was lower in O+ individuals (ANOVA, *p*<0.01) compared with O− individuals (*p*<0.01, 95% CI −2.05, −0.43) and those with no DPN (*p*<0.01, 95% CI −5.2, −3.4). The MDT was also significantly different in O− individuals compared to those with no DPN (*p*<0.01, 95% CI 2.3, 3.8). The severity of neuropathic pain was significantly higher in O+ individuals compared with O− individuals (*t* test, *p*<0.01).

### Structural neuroimaging analysis

**Fig. 2:**
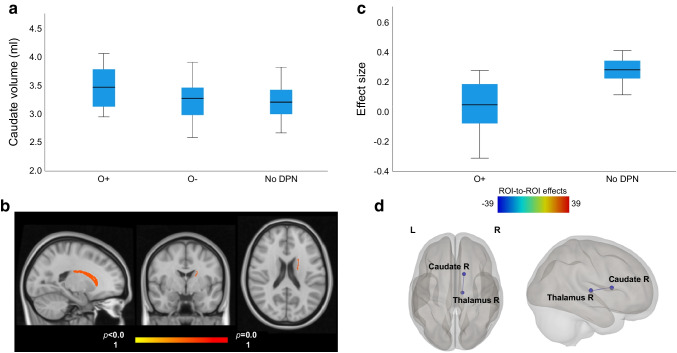
(**a**) Box and whisker plot of caudate volume (ml) analysed using FreeSurfer. ANOVA, *p*=0.03 (O+ individuals vs O− individuals: *p*=0.03, 95% CI 25.0, 438.0; O+ individuals vs those with no DPN: *p*=0.01, 95% CI 76.2, 524.1); whiskers extend from the smallest and largest values 1.5×IQR. (**b**) Left caudate volume in O+ individuals compared with O− individuals by FSL-FIRST analysis (O+ >O−, *p*<0.05). From left to right: sagittal, coronal and axial views. (**c**) Box and whisker plot showing the effect size for functional connectivity differences between caudate and thalamus in O+ individuals and those with no DPN (uncorrected *t* test *p*=0.034); whiskers extend from the smallest and largest values 1.5×IQR. (**d**) Difference in functional connectivity between O+ individuals and those with no DPN. ROI spheres correspond to the centre of the region of the atlas used in the CONN toolbox software. Left image, axial view; right image, sagittal view. The coloured key indicates the coloured dots and lines and shows the seed level correction, referring to strength of functional connectivity correlation/anti-correlation between ROIs, L, left; R, right

Global measures of segmented brain volumes were not significantly different between the three groups (Table 2). The caudate volume was significantly greater in O+ individuals (mean caudate volume 3.5±0.4 ml; ANOVA, *p*=0.03) compared with O− individuals (3.2±0.4 ml; *p*=0.03, 95% CI 25.0, 438.0) and those with no DPN (3.2±0.3 ml; *p*=0.01, 95% CI 76.2, 524.1) (Fig. 2a). Although O+ individuals had a greater volume of the putamen (*t* test, *p*=0.130), amygdala (*p*=0.364) and nucleus accumbens (*p*=0.111), the comparison with O− individuals was not statistically significant. There were also no differences in cortical brain volumes or vertices.

**Table 2:** Global and regional brain parameters, stratified by group

Brain parameter	O+ individuals (*n*=18)	O− individuals (*n*=39)	No DPN (*n*=25)	*p* value
Global				
Total brain volume (l)	1.06±0.1	1.01±0.1	1.05±0.1	0.18
Cortical volume (ml)	413.3±42.1	391.2±45.3	400.5±43.4	0.24
Total cortical white matter volume (ml)	472.2±61.4	446.2±50.6	445.2±61.3	0.30
Subcortical grey volume (ml)	53.3±4.5	51.4±4.7	51.9±4.5	0.37
Total grey volume (ml)	561. 3±50.0	532.6±55.2	542.0±54.0	0.21
Regional				
Mean S1 thickness (mm)	1.86±0.1	1.87±0.1	1.9±0.1	0.97
Mean M1 thickness (mm)	2.29±0.1	2.27±0.2	2.4±0.1	0.10
Mean insula thickness (mm)	2.8±0.2	2.8±0.2	2.9±0.2	0.54
Mean ACC thickness (mm)	2.6±0.2	2.5±0.2	2.6±0.3	0.63
Mean thalamic volume (ml)	6.2±0.7	6.5±0.8	6.3±0.8	0.54
Mean caudate volume (ml)	3.5±0.4	3.2±0.4	3.2±0.3	0.03
Mean putamen volume (ml)	4.9±0.4	4.8±0.6	4.8±0.5	0.49
Mean amygdala volume (ml)	1.5±0.2	1.5±0.2	1.5±0.2	0.90
Mean nucleus accumbens volume (ml)	2.8±0.2	2.6±0.4	2.6±0.3	0.42

Data are presented as means ± SD and compared using ANOVA

ACC, anterior cingulate cortex; M1, primary motor cortex; S1, primary somatosensory cortex

FSL-FIRST analysis confirmed a significant difference in caudate volume (O+ > O−), suggesting greater caudate volume in O+ individuals compared with O− individuals (Fig. 2b). There was no difference in the O− > O+ analysis. Voxel-based morphometry analysis was performed, and no significant difference was detected on group analysis between any of the three groups.

There was a significant correlation between caudate volume and age (Pearson’s *r*=−0.265, *p*=0.015) and severity of neuropathic pain (Pearson’s *r*=0.381, *p*<0.001). There was no correlation with any other measured parameter, including neurophysiological measures.

### Functional neuroimaging analysis

A total of four O+ individuals had not undergone rs-fMRI, therefore 14 participants from each group were included in the functional neuroimaging analysis (O− individuals and those with no DPN were matched to O+ individuals) (see Fig. 1). rs-fMRI functional connectivity ROI-to-ROI analysis showed a significantly lower functional connectivity between the caudate and thalamus in O+ individuals (Fig. 2c, d; *r* β = −0.24, seed-level correction −3.9, *p*_FDR_ ≤0.05) compared to those with no DPN, whereas there was significantly higher functional connectivity between the right putamen and left insular cortex in O− individuals compared to those with no DPN (*r* β = 0.19, seed-level correction −3.45, *p*_FDR_ ≤0.05). There were no significant differences in functional connectivity between O+ individuals and O− individuals on ROI-to-ROI analysis. However, seed-to-voxel analysis using the right caudate as the seed indicated a significantly lower functional connectivity in O+ individuals compared with O− individuals in a cluster encompassing the superior frontal gyri bilaterally (MNI152 coordinates −8, +22, +62; cluster size 238; *p*_FDR_=0.03; ESM Fig. 1).

## Discussion

In this novel multimodal neuroimaging study, we demonstrate the presence of structural and functional brain alterations related to the caudate nucleus in people with painful DPN taking long-term opioid therapy. There was a greater caudate volume, demonstrated using two complementary structural analysis techniques (FreeSurfer and FSL-FIRST). Moreover, there was lower caudate nucleus to thalamic functional connectivity in people with painful DPN taking long-term opioid therapy compared to those with diabetes but without painful DPN. Using seed-to-voxel analysis, there was also lower caudate to superior frontal gyri connectivity in people with painful DPN taking long-term opioid therapy, compared to those with painful DPN who were not taking long-term opioid therapy.

To our knowledge, this is the first study to examine structural and functional parameters in the brain due to prescription opioid use for painful DPN. The effects on the brain of opioids such as heroin taken for recreational use have been well studied. However, studies of the effects of prescription opioids on brain structure and function have largely focused on lower back pain [12, 13]; our study extends these findings to painful DPN. Younger et al performed longitudinal imaging at baseline and 1 month after commencement of oral morphine in ten individuals with back pain, and found dose-correlated decreases in the volume of the amygdala and increases in the volume of the hypothalamus, inferior frontal gyrus, ventral posterior cingulate and caudal pons, while those prescribed placebo had no morphological alterations over time [12]. Murray et al performed structural and functional brain analysis in 11 people prescribed opioids for chronic back pain in comparison with 30 participants with chronic back pain but not prescribed opioids and 30 healthy controls [13]. Although patient age was significantly higher in the group prescribed opioids, potentially acting as a confounding factor, individuals prescribed opioids had a reduced volume of the nucleus accumbens and thalamus, and lower resting-state activity for the nucleus accumbens. Now, our study demonstrates opioid-related brain alterations in a distinct chronic pain condition, painful DPN, providing further insights into the neural effects of long-term opioid use in patients with neuropathic pain. Understanding the impact of opioids on the brain is important, as the available medications for painful DPN are limited in efficacy [2] and opioids remain a commonly prescribed treatment [9].

The caudate nucleus is a paired subcortical structure, lying deep inside the brain near to the thalamus. It is involved in the planning of movement, and learning, memory, motivation and emotion. The caudate is activated during acute pain and is potentially involved in reducing the affective component of pain in normal physiology [21]. We previously found that caudate volume was not different in people with painful DPN compared to patients with diabetes without DPN, healthy controls and people with painless DPN [14]. Moreover, other studies of chronic pain conditions have demonstrated a reduction in caudate volume [22] and reduced functional connectivity of the caudate to other brain regions involved in pain processing [11, 23]. Therefore, the finding that opioids lead to the brain changes in this study would be consistent with other research in the field; however, potential confounding factors and the possibility of reverse causation (i.e. those with greater caudate nucleus volume experiencing more pain, therefore requiring opioid treatments) cannot be excluded due to the cross-sectional nature of the study. Prospective studies are required to confirm the causality of our findings.

Structural and functional alterations in the caudate are associated with opioid abuse and opioid use disorders [24, 25]. Functional MRI studies have demonstrated alterations in caudate blood oxygen level-dependent signals associated with heroin-related cues, and increases in resting-state functional connectivity of the caudate to other brain regions [24]. Moreover, a longitudinal study demonstrated enlargement of the caudate in patients with opioid use disorder associated with prescription of an injectable opioid agonist (diacetylmorphine) for 9 years as an alternative to illicit drug use [25]. Enlargement of the caudate may suggest alterations in memory/habit learning circuits or reward-seeking/stimulus–response systems associated with heroin abuse and opioid use disorder [25]. There is therefore biological plausibility that the structural and functional brain alterations found in this study are due to opioid prescription.

We also demonstrate a lower functional connectivity between the caudate and thalamus associated with long-term opioid use. The thalamus and caudate are known to be functionally linked in the nigrostriatal pathway [26]. This is a dopaminergic pathway connecting the dorsal striatum (i.e. caudate and putamen) and substantia nigra pars compacta, with axons of the latter extending collaterals to the thalamus. The nigrostriatal system is involved in reward function and has been implicated in the habit-forming properties of addiction [27, 28]. Altered nigrostriatal activity accompanying drug exposure has been hypothesised to produce a state of feedback insensitivity promoting altered behaviours such as the stereotypic, rigid behavioural patterns contributing to relapse [29]. Consistent with this, and our findings, rodent models of alcohol addiction demonstrate a hypodopaminergic state in the nigrostriatal pathway related to compulsive-like alcohol use [30], and clinical studies have demonstrated reduced functional connectivity between the thalamus and caudate [31, 32] and greater caudate volume in smokers compared with non-smokers [32, 33]. Moreover, dopaminergic pathways show reduced functional connectivity in heroin users [34, 35]. Further research should explore whether reduced functional connectivity between the caudate and thalamus in long-term opioid users is causally linked to the development of additive behaviours or relapse. Longitudinal studies examining changes in connectivity over time in individuals initiating or discontinuing opioid use could clarify the role of connectivity in the progression or resolution of addiction. Additionally, neuroimaging studies assessing dopaminergic activity in the nigrostriatal pathway in opioid users, together with comparisons to other substance use disorders, would deepen our understanding of how disrupted connectivity contributes to rigid behavioural patterns and compulsivity. Finally, exploring interventions that could potentially restore functional connectivity in the nigrostriatal pathway, such as targeted pharmacotherapies or behavioural therapies, may provide insights into treatment approaches that reduce addiction-related rigid behaviours and relapse risk.

This study also demonstrated lower functional connectivity between the caudate and superior frontal gyrus in O+ individuals compared with O− individuals by seed-to-voxel analysis. The superior frontal gyrus is a part of the prefrontal cortex, which has a number of processes that are fundamental for neuropsychological function, encompassing emotion, cognition and behaviour [36]. Disruption in this connectivity may reflect changes in the mesolimbic pathway, a dopaminergic circuit that is crucial for reward processing and impulse control. Dysfunction of this pathway in opioid users implies that prescription opioids could contribute to alterations in brain circuits associated with addiction, potentially heightening susceptibility to compulsive behaviours and impairing decision-making. These results underscore the need to evaluate the impact of long-term opioid use on neurocognitive function and investigate potential interventions to mitigate these disruptions [37].

It is increasingly recognised that the risk of opioid prescription outweighs the benefits, with a growing body of evidence finding negative consequences associated with opioid use [4]. Preliminary findings suggest potential differences in brain function and structure after 6 months of opioid treatment, although causality cannot be inferred from this cross-sectional study. It is also not known whether these alterations are reversible. Dependence syndromes such as opioid use disorder are notoriously difficult to treat and have a poor prognosis [38]. The concerning findings of this study further underscore the urgent need to enforce stricter prescribing standards. Recent guidelines have recommended against the use of opioid therapy [39]. If opioid therapy is being considered, a risk assessment is critical to providing the best possible patient-centred outcome while avoiding unnecessary opioid exposure [40]. It is important that regulatory changes be enacted to prioritise patient safety and consider the long-term impacts of opioid therapies.

There are several strengths to this study. First, the study performed multimodal structural brain analysis in all participants, and rs-fMRI and structural brain imaging in most study participants. This allowed examination of structural and functional brain alterations in our cohort, and use of the three structural analysis techniques allowed us to explore in detail the potential volumetric changes associated with long-term opioid use. Moreover, the sample size is consistent with other studies within the field [12, 13]. Also, other studies assessing the impact of brain alterations associated with opioid use often use healthy participants as the control group [24], our study included a comparator group (O− individuals) and a disease control group (no DPN), with the groups being matched for age, sex and type of diabetes. Scanning was performed after participants had stopped analgesia for at least 48 h. This is necessary to control the pharmacological effect on neuroimaging measures; however, the transient effects of washout are unlikely to alter the study conclusions. Study limitations include its cross-sectional nature, which means that we cannot determine the causality of our results. There were also baseline differences in clinical factors, and larger studies should aim for more balanced groups with robust statistical approaches (e.g. propensity score matching) in order to validate our findings. Also, due to the fact this was a database study, we did not have detailed information on opioid adverse events, such as opioid use disorder, nor details on opioid dosing (e.g. morphine-equivalent doses of opioids) or other non-drug related addictions (e.g. gaming, gambling etc.), and also other potential confounding factors such as physical activity that could alter brain structure/function. Moreover, although we knew that all individuals had been prescribed opioids for more than 6 months (and excluded those with an unconfirmed duration or duration of less than 6 months), we did not have data on the exact duration of opioid use. This study opens new research avenues, and future studies will need to perform more detailed characterisation of addictive behaviour, opioid prescription and opioid adverse events to explore the interaction of brain changes and opioid prescription. Ideally, prospective studies should be performed that longitudinally examine the changes within the brain in people with painful DPN upon prescription of opioids.

In summary, we demonstrate for the first time in patients with painful DPN that there are structural and functional alterations within the caudate nucleus in people with long-term prescription opioid use. The caudate nucleus is a key brain region that is associated with the dopaminergic pathways implicated in addiction/substance use disorders. Thus, although the study was cross-sectional, and causality cannot be inferred, the alterations shown in this study may reflect alterations in the reward system due to long-term opioid prescription. Future studies are needed to longitudinally examine the interaction between chronic pain, opioid adverse effects (opioid use disorder/tolerance/withdrawal/addictive behaviour) and changes in dopaminergic pathways. This study may have important clinical implications, as the structural or functional alterations in dopaminergic pathways may represent neuroimaging biomarkers that could be used to diagnose and monitor the negative consequences of prescription opioid use [13, 25]. Further validation of these measures is required, including studies with larger and more diverse cohorts to assess their robustness and generalisability. Additionally, longitudinal research is needed to evaluate their predictive value over time before they can be considered as potential biomarkers of prescription opioid use.

## Supplementary Information

Below is the link to the electronic supplementary material.

ESM (PDF 176 KB)

## Data Availability

The datasets generated and/or analysed during the current study are available from the corresponding author upon reasonable request.

## Abbreviations

CDT: Cold detection threshold
CPT: Cold pain threshold
DMA: Dynamic mechanical allodynia
DPN: Diabetic peripheral neuropathy
FDR: False discovery rate
HPT: Heat pain threshold
MDT: Mechanical detection threshold
MPS: Mechanical pain sensitivity
MPT: Mechanical pain threshold
O−: Individuals with painful DPN with no evidence of opioid prescription within the last 12 months
O+: Individuals with painful DPN with long-term opioid prescription, defined as >6 months prescription
PPT: Pressure pain threshold
QST: Quantitative sensory testing
ROI: Region of interest
rs-fMRI: Resting-state functional MRI
TSL: Thermal sensory limen
VDT: Vibration detection threshold
WDT: Warm detection threshold

## Authors’ relationships and activities

ST has received lecture honoraria from Wörwag Pharma, Pfizer, Novo Nordisk, Merck, EVA Pharma, Hikma Pharmaceuticals, Grünenthal, Abbott Laboratories, AstraZeneca, Trigocare International, and Procter & Gamble and is on the medical advisory board of Bayer. GS has received honoraria from Procter & Gamble and Eli Lilly. DS is a member of the editorial board of *Diabetologia*. The remaining authors declare that there are no relationships or activities that might bias, or be perceived to bias, their work.
