# Supplementary material for: Beyond pain relief: the effects of chronic opioid use on brain structure and function in diabetic neuropathy—a multimodal neuroimaging study
Source: Diabetologia. 2025 Oct 6;68(12):2867–78. doi: 10.1007/s00125-025-06529-w (PMC12594651; doi:10.1007/s00125-025-06529-w)
Supplement: Supplementary file 1 — ESM (PDF 176 KB) [file 125_2025_6529_MOESM1_ESM.pdf]

Electronic supplementary materials

Methods

MRI Acquisition and Analysis

High-resolution, three-dimensional cerebral MRI scans were acquired at 3 T (Ingenia, Philips Healthcare, Best, the Netherlands; Intera, Philips Healthcare, Best, the Netherlands). Scanning parameters for the T1-weighted magnetisation prepared rapid acquisition gradient echo sequence were as follows: flip angle=8°, echo time (TE)=3.9ms; repeat time (TR)=8.3ms and reconstructed voxel size=0.94×0.94×1mm3. For the 3 T Intera the parameters were as follows: flip angle=8°; echo time (TE)=3.3ms; repeat time (TR)=7.3ms and reconstructed voxel size =0.83x0.83×0.9×1mm^3. The rs-fMRI sequence was obtained after participants were asked to remain as still as possible in the scanner and while they were fixated on a cross to avoid brain wandering. The scan was a a T2*-weighted pulse sequence with the following parameters: TR 2600ms, TE 3.5ms, in-plane pixel dimensions 1.8mm x 1.8mm, contiguous trans-axial slices 4mm orientated in the oblique axial plane. We acquired 150 volumes with a total scan time of 6.5 minutes.

Cortical reconstruction and volumetric segmentation were performed with the Freesurfer software (https://surfer.nmr.mgh.harvard.edu) to determine cortical thickness (mm), volume (ml) and vertices. Regions of interest were chosen in regions related to somatomotor function (primary somatosensory [S1] and motor [M1] cortex, insular cortex, anterior cingulate [ACC] gyrus, thalamus) and key regions involved in prescription opioid dependence (putamen, amygdala, nucleus accumbens and caudate nucleus).

This processing includes motion correction and averaging [1] of volumetric T1 weighted images, removal of non-brain tissue using a hybrid watershed/surface deformation procedure [2], affine-registered to the Talairach atlas [2, 3], intensity normalization, tessellation of the grey matter white matter boundary, automated topology correction [4, 5], and surface deformation following intensity gradients to optimally place the grey/white and grey/cerebrospinal fluid borders at the location where the greatest shift in intensity defines the transition to the other tissue class [6, 7]. Surface based maps are created from the intensity and continuity information from the entire 3D MR volume in segmentation and deformation procedures to produce representations of cortical thickness (mm), calculated as the closest distance from the grey/white boundary to the grey/CSF boundary at each vertex on the tessellated surface [4].

Voxel based morphometric analysis was performed according to a set of prior guidelines [8]. Both pre-processing and statistical analysis of anatomical data were carried out using the FMRIB Software Library (FSL, version 6.0.3). Voxel-based morphometry (VBM), an optimised voxel-based morphometry analysis toolbox implemented in FSL was used as this approach is validated, operator-independent and efficient [9]. After an initial weak bias field correction, structural images were brain-extracted and grey matter-segmented before being registered to the MNI 152 standard space using non-linear registration [10]. Next a study specific grey matter template was created by averaging and flipping the images along the x-axis creating a left-right symmetric. All native grey matter images were then non-linearly registered to this study-specific template and "modulated" to correct for local expansion (or contraction) due to the non-linear component of the spatial transformation. A smoothing kernel of 3mm full-width at half-maximum (FWHM) was applied to the segmented gray matter images to enhance statistical robustness and account for inter-subject variability.

Voxel wise general linear modelling was applied using permutation-based (5,000 permutations) nonparametric testing. Clusters of significance were identified using the threshold-free cluster

enhancement method [11], taking family-wise error rate–corrected $p < 0.05$. The family-wise error multiple comparison correction is based on Bonferroni method and controls the likelihood of false positive findings in analysis. Probabilistic anatomical descriptors were determined using FSL atlas query avoiding labelling bias, and cluster peak information was extracted using the FSL cluster tool for voxels that had survived multiple comparison correction.

We conducted a region of interest (ROI) analysis using the CONN toolbox to examine functional connectivity patterns. Preprocessing of fMRI data included realignment, slice-timing correction, normalization to MNI space, and spatial smoothing. ROIs were defined using the Automated Anatomical Labeling (AAL) atlas and literature-specific coordinates. We extracted the mean BOLD signal from each ROI, calculated Pearson correlation coefficients between ROI pairs, and applied Fisher Z-transformation for normality. Group-level statistical analyses, including one-sample and two-sample *t*-tests, were performed to identify significant within-group and between-group connectivity patterns, with False Discovery Rate (FDR) correction applied for multiple comparisons. Results were visualized on a 3D brain template, highlighting significant connections to interpret functional connectivity networks relevant to the study's hypotheses.

Results

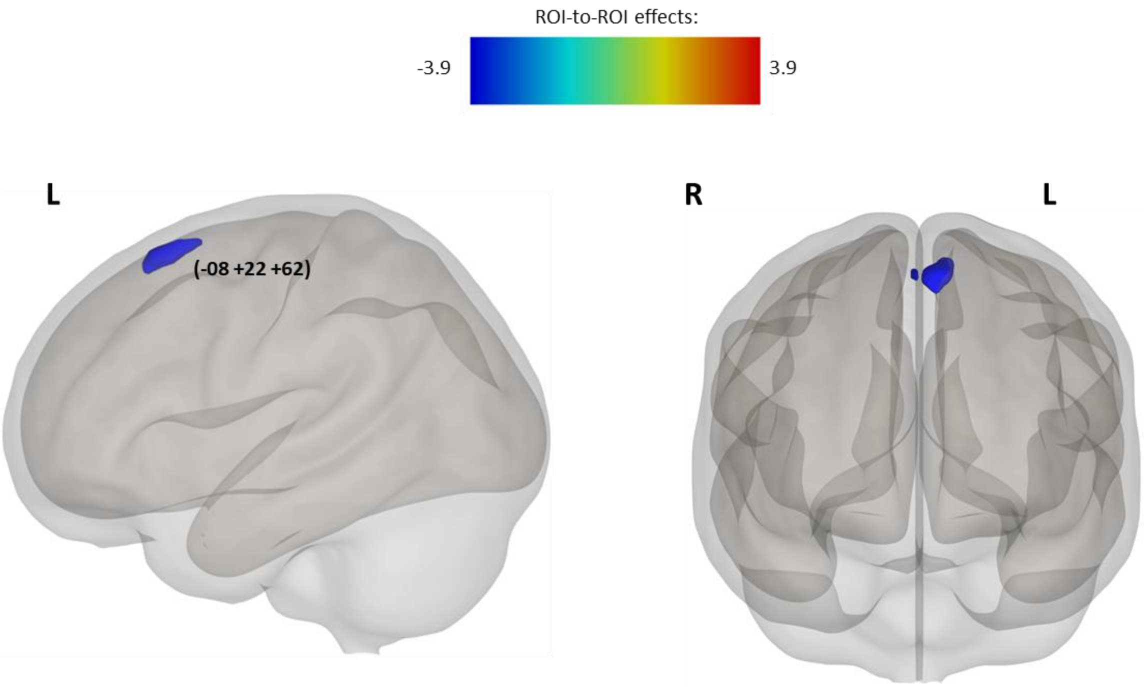

ESM Figure 1. Seed-to-voxel analysis using the right caudate as the seed in O+ compared to O- (MNI 152 co-ordinate, -8, +22, +62; cluster size 238; p_{FDR}=0.03), sagittal and coronal views.

[1] Reuter M, Rosas HD, Fischl B (2010) Highly accurate inverse consistent registration: a robust approach. Neuroimage 53(4): 1181-1196. 10.1016/j.neuroimage.2010.07.020
[2] Ségonne F, Dale AM, Busa E, et al. (2004) A hybrid approach to the skull stripping problem in MRI. Neuroimage 22(3): 1060-1075. 10.1016/j.neuroimage.2004.03.032

[3] Fischl B, Salat DH, Busa E, et al. (2002) Whole brain segmentation: automated labeling of neuroanatomical structures in the human brain. Neuron 33(3): 341-355. 10.1016/s0896-6273(02)00569-x
[4] Fischl B, Liu A, Dale AM (2001) Automated manifold surgery: constructing geometrically accurate and topologically correct models of the human cerebral cortex. IEEE Trans Med Imaging 20(1): 70-80. 10.1109/42.906426
[5] Ségonne F, Pacheco J, Fischl B (2007) Geometrically accurate topology-correction of cortical surfaces using nonseparating loops. IEEE Trans Med Imaging 26(4): 518-529. 10.1109/TMI.2006.887364
[6] Dale AM, Fischl B, Sereno MI (1999) Cortical surface-based analysis. I. Segmentation and surface reconstruction. Neuroimage 9(2): 179-194. 10.1006/nimg.1998.0395
[7] Fischl B, Dale AM (2000) Measuring the thickness of the human cerebral cortex from magnetic resonance images. Proc Natl Acad Sci U S A 97(20): 11050-11055. 10.1073/pnas.200033797
[8] Ridgway GR, Henley SM, Rohrer JD, Scahill RI, Warren JD, Fox NC (2008) Ten simple rules for reporting voxel-based morphometry studies. Neuroimage 40(4): 1429-1435. 10.1016/j.neuroimage.2008.01.003
[9] Ashburner J, Friston KJ (2000) Voxel-based morphometry--the methods. Neuroimage 11(6 Pt 1): 805-821. 10.1006/nimg.2000.0582
[10] Rinne-Albers MA, Pannekoek JN, van Hoof MJ, et al. (2017) Anterior cingulate cortex grey matter volume abnormalities in adolescents with PTSD after childhood sexual abuse. Eur Neuropsychopharmacol 27(11): 1163-1171. 10.1016/j.euroneuro.2017.08.432
[11] Smith SM, Nichols TE (2009) Threshold-free cluster enhancement: addressing problems of smoothing, threshold dependence and localisation in cluster inference. Neuroimage 44(1): 83-98. 10.1016/j.neuroimage.2008.03.061
